# Caregiver distress: A mixed methods evaluation of the mental health burden of caring for children with bladder exstrophy

**DOI:** 10.3389/fped.2022.948490

**Published:** 2022-10-14

**Authors:** Katelyn A. Spencer, Jaishri Ramji, Pooja Unadkat, Iqra Nadeem, Parth A. Lalakia, Jay Shah, Pramod P. Reddy, Douglas A. Canning, Joao Pippi-Salle, Paul Merguerian, Anjana Kundu, Dana A. Weiss, Aseem R. Shukla, Rakesh Joshi, Jennifer R. Frazier

**Affiliations:** ^1^Department of General Surgery, Division of Urology, Children’s Hospital of Philadelphia, Philadelphia, PA, United States; ^2^B. J. Medical College and Civil Hospital, Ahmedabad, India; ^3^Department of Surgery, Division of Pediatric Urology, Cincinnati Children’s Hospital, Cincinnati, OH, United States; ^4^Sidra Medical and Research Center, Doha, Qatar; ^5^Department of General Surgery, Division of Urology, Seattle Children’s Hospital, Seattle, WA, United States; ^6^Department of Anesthesiology and Pain Medicine, University of Rochester, Rochester, NY, United States

**Keywords:** bladder exstrophy-epispadias complex (BEEC), caregiver distress, quality of life, low- and middle-income countries (LMIC), stigma

## Abstract

**Introduction:**

Caring for children with bladder exstrophy-epispadias complex (BEEC) exacts a long-term emotional toll on caregivers. Previous studies leave a gap in understanding the impact that caring for a child with BEEC has on caregivers in low- and middle-income countries (LMIC). We hypothesize that families and caregivers experience psychological distress that has long gone unaddressed.

**Materials and methods:**

From 2018 to 2020, researchers conducted a multi-method evaluation of caregiver distress with participants recruited as part of the annual International Bladder Exstrophy Collaboration based in Ahmedabad, Gujarat, India. In 2018, pilot data was collected through cognitive interviews. In 2019, researchers conducted structured interviews predicated on themes from the previous year, which subsequently prompted formal mental health screenings in 2020. Caregivers who reported suicidal thoughts were immediately referred for intervention.

**Results:**

In 2018, caregivers described the primary source of stigma arose from their village (*n* = 9, 26.5%). Caregivers also identified long-term concerns (*n* = 18, 52.9%), including future fertility and marital prospects, as sources of anxiety. In 2019, caregivers substantiated preliminary findings with the primary source of anticipated (*n* = 9, 31%) and experienced (*n* = 19, 65.5%) stigma again stemming from their communities. Both cohorts identified the collaboration as a positive source of support (*n* = 23, 36.5%). In 2020, caregivers stated decreased emotional wellbeing as number of subsequent repairs increased (*n* = 54, 75%, *p* = 0.002). Caregivers of children who underwent initial surgery within 5 years of screening reported higher anxiety (*n* = 46, 63.8%) and this was exacerbated as the number of subsequent repairs increased (*p* = 0.043).

**Conclusion:**

Complex, long-term course of care, including additional surgeries, significantly impacts caregiver distress in the LMIC setting. Screening for caregivers of children with complex congenital anomalies, like BEEC, should be an essential element of any comprehensive effort to alleviate the global burden of disease.

## Introduction

Similar to other children with significant anatomic defects, bladder exstrophy-epispadias complex (BEEC) patients are at a greater risk of psychosocial adjustment issues, including suicide ideation and behaviors (1), depression, and anxiety when compared to physically healthy children (2). The caregivers of these children also experience increased rates of stress that are comparable to the experiences of parents of children with other significant diagnoses, like type 1 diabetes (3). Specifically, BEEC and its associated incontinence causes stigma which can result in the isolation of a patient and their caregiver from family and community members (4). This increases the complexity of care that these patients need in order to achieve long-term wellbeing and maximize health-related quality of life.

The experienced and anticipated stigma (5) that these children and their caregivers can experience exists in all settings, but they are further complicated in low- and middle-income countries (LMICs) where 94% of congenital anomalies occur (6). Patients in these settings generally have less access to mental and physical health care (7). And, importantly for BEEC patients and caregivers, LMICs have less access to urinary absorptive clothing such as disposable diapers (4). Specifically, in India where the International Bladder Exstrophy Consortium operates, patients overwhelmingly live in close, multigenerational arrangements (8). This creates additional challenges for patients and their caregivers as the consequences of incontinence are amplified within close quarters.

The intensive surgical and medical management required to achieve anatomic integrity and future continence of children with BEEC may also be a contributing factor to caregiver and patient distress. The International Bladder Exstrophy Consortium (9), conceived in 2009 to partly address a large burden of unmet surgical need in India, utilizes the complete primary repair of exstrophy (CPRE) in an effort to minimize the number of subsequent repairs. But, the post-operative period can still be complicated by numerous events that could necessitate additional operations including penopubic fistula, bladder outlet obstruction, hypospadiac meatus at closure, and bladder dehiscence. These complications and the necessity for future operations are also impacted by the age at presentation for surgery and whether the patient requires a primary or redo repair after a failed initial closure at another location. We hypothesize that caregivers of BEEC patients will identify themes of stress, anxiety, and stigma within their lives and that higher numbers of subsequent surgeries contribute to higher levels of caregiver distress and anxiety.

## Materials and methods

A sustainable, long-term, and mutually beneficial collaboration between academic research centers from high income countries (HICs) and a tertiary hospital in Ahmedabad, Gujarat, India with appropriate capacity was created in 2009 to provide care for BEEC patients (9). Overtime, the collaboration has grown to include Cincinnati Children’s Hospital, Children’s Hospital of Philadelphia, Seattle Children’s Hospital, surgeons from Sidra Medical Center in Doha, Qatar, and a pediatric anesthesiologist with specialization in pediatric pain management. The annual collaboration takes place at a government funded, public hospital that serves a geographic catchment area of 60 million people. The collaboration recalls all of the patients annually, in addition to new patients, to be evaluated by a team of surgeons that are consistent from year to year. At this time, progress studies, including urodynamic evaluation, cystogram, ultrasound, and DMSA scans are performed. Surgical and follow-up plans are made for all patients.

The increased importance of long-term wellbeing and the impact of stigma on health-related quality of life spurred the collaboration to begin evaluating more holistic measures of post-operative success to assess gaps of care within the program. From 2018 to 2020, researchers conducted a multi-method evaluation of caregiver distress with participants recruited from the annual surgical collaboration. Sampling of patients was performed using a convenience sample of all returning participants of the cohort. Data was collected throughout the collaboration as time allowed. Each instrument used was independently forward and reversed translated in two regional languages and reviewed by expert members of the research team to screen for translation errors. Participants were surveyed by a bicultural and bilingual interpreter independent of the surgical team. Surgical history was collected from the medical team. IRB approval was provided by the Institutional Ethics Committee of the Civil Hospital, Ahmedabad and BJ Medical College. Written informed consent to participate in this study was provided by the patient’s caregiver and/or the participant’s legal guardian/next of kin.

In 2018, researchers collected pilot data through cognitive interviews using two existing quality of life questionnaires as a guide when creating the interview instrument: The Pediatric Urinary Incontinence Quality of Life tool (10) and the Chronic Illness Anticipated Stigma Scale (5). Participants included caregivers and patients who were awaiting initial repair of BEEC or returning for their annual follow-up visit. Information documented included patient demographics, questions for doctors, and perceptions of stigma.

In 2019, researchers returned to the annual surgical collaboration to conduct more focused and structured interviews based on the themes that emerged from 2018. All interviews were conducted in Gujarati and Hindi, recorded, forward and reverse translated by two unique individuals, transcribed, coded, and analyzed using NVivo. Data analysis and collection followed a grounded theory through an iterative process (11). Codes and themes were inductively developed using descriptive and *in vivo* coding techniques. Descriptive coding summarizes the topic of a brief section of the interview while *in vivo* coding uses the actual language of the qualitative data to produce a code (12). The codes were used to analyze the data and extrapolate themes from the interviews. Before finalizing the codebook, subthemes were identified until code and meaning saturation was achieved. Code saturation describes when no new themes have been identified by participants, and meaning saturation is when no new dimensions of a theme have been identified by participants.

These structured interviews reinforced the pilot data and motivated researchers to conduct formal depression and anxiety screenings in 2020 using well-validated quantitative measures of mental health indicators and health related quality of life. Participants were administered the Beck Depression Inventory (13), Beck Anxiety Inventory (14), and RAND 36-item Health Survey (15) to evaluate distress. These inventories are well-validated measures that have test-retest reliability and are widely used to detect and assess the intensity of anxiety and depression in patient populations. The RAND 36-Item questionnaire was administered to 73 patients. This questionnaire is one of the most widely used health-related quality of life questionnaires, and it assesses physical functioning, role limitations caused by physical health problems, role limitations caused by emotional problems, social functioning, emotional wellbeing, energy/fatigue, pain, and general health perceptions (15). Caregivers who reported suicidal thoughts were immediately referred to a visiting counselor. All data analysis was performed by IBM’s SPSS 28.0.1.

## Results

The findings of patient and caregiver interviews, including prominent themes and illustrative quotes, are presented in Tables 1, 2, respectively. In 2018, 34 participants were interviewed. Caregivers reported protective factors against stigma and anxiety including living with supportive families (*n* = 28, 82.4%) and interacting with (*n* = 8, 23.5%) or anticipating cooperative school administrators and employers (*n* = 4, 11.8%). Families reported that the majority of experienced (*n* = 8, 23.5%) and anticipated (*n* = 1, 2.9%) stigma arises from their village. Over half (*n* = 18, 52.9%) of caregivers report anxiety around the post-operative experience including concerns about future continence (*n* = 14, 41.2%) and marital prospects (*n* = 6, 17.6%). However, families report that they have a positive outlook for the future (*n* = 16, 47.1%) and the collaboration has helped them create a network of support they do not encounter in their hometowns (*n* = 7, 20.6%). Additionally, almost half (*n* = 13, 38.2%) expressed a high degree of trust in the medical team.

**TABLE 1:** Qualitative interview themes.

Themes	Total		2018 Cohort (*n* = 34)		2019 Cohort (*n* = 29)	

Classmates and friends						
Anticipated stigma	13	21%	3	8.8%	10	34.5%
Experienced stigma	8	13%	4	11.8%	4	13.8%
Anticipated support	4	6%	2	5.9%	2	6.9%
Experienced support	9	14%	2	5.9%	7	24.1%
**Collaboration benefits**						

Found support and friends	23	37%	7	20.6%	16	55.2%
Gained perspective	11	17%			11	37.9%
Trust the doctors	25	40%	13	38.2%	12	41.4%
**Family**						

Anticipated stigma	5	8%	2	5.9%	3	10.3%
Experienced stigma	22	35%	10	29.4%	12	41.4%
Anticipated support	0	0%				
Experienced support	44	70%	28	82.4%	16	55.2%
Emotional	13	21%			13	44.8%
Financial	3	5%			3	10.3%
Physical	8	13%			8	27.6%
**Future concerns**						

Future fertility	18	29%	2	5.9%	16	55.2%
Ability to be independent	7	11%			7	24.1%
Future continence	28	44%	14	41.2%	14	48.3%
General quality of life	10	16%			10	34.5%
Marital prospects	25	40%	6	17.6%	19	65.5%
None	7	11%			7	24.1%
Genital cosmesis	4	6%	3	8.8%	1	3.4%
Positive outlook	28	44%	16	47.1%	12	41.4%
**School administrators and bosses**						

Anticipated stigma	4	6%	1	2.9%	3	10.3%
Experienced stigma	5	8%	1	2.9%	4	13.8%
Anticipated support	10	16%	4	11.8%	6	20.7%
Experienced support	17	27%	8	23.5%	9	31.0%
**Surrounding community**						

Anticipated stigma	10	16%	1	2.9%	9	31.0%
Experienced stigma	27	43%	8	23.5%	19	65.5%
Anticipated support	1	2%	1	2.9%		
Experienced support	12	19%	3	8.8%	9	31.0%

**TABLE 2:** Illustrative quotes from patients and caregivers.

Community	
“A lot of families say that they don’t like “the urinating child,” so we hesitate to take our child anywhere. Whenever our child goes to their house and sits on their couch, they get disgusted and become hesitant towards him sitting there.”	
“We got our daughter engaged. The in-laws would come visit our house often and one time we visited their house as well. My child was playing around and sat down on the mattress and urinated on it. My daughter’s to-be mother-in-law said really mean and bad words to us because of that. I got really emotional because my daughter’s engagement got broken off because of that.”	
“We are working so hard for her right now that our daughter becomes normal just like other children. We don’t want her to feel any shame from the society.”	
“They call her “dirty [patient name]”. They kept saying that she smells bad and wouldn’t let her play with other kids. They also told me, what kind of daughter have you given birth to, telling me that I have done bad things in life and this is the result of that.”	
“People do talk about whether she will be able to get married when she grows up and also question what we will do if her urine leakage won’t stop. All the community members say this on my face and behind my back as her condition is very obvious and right in front of us.”	
“The community members don’t say anything to our faces, however, they do talk behind our back. Others have told us that community members still talk about his conditions in our absence. We feel bad about their talks, however, we can’t do anything about it so we just ignore them.”	

**Future concerns**	**Classmates**

‘We are not worried about anything right now. After Dr. Reddy’s presentation, I am not that concerned about his condition or future anymore. Before that we were worried about marriage and having kids in the future after all the surgeries get done. No, I don’t have any questions right now.”	“However, the children at school do tease him by calling him names. People in the neighborhood also tease him, and he comes home crying. His mom gets frustrated and goes to fight with their mothers. This causes disturbance and arguments with our neighbors.”
“We also worry and hope that she won’t take any bad steps of harming herself because of her physical condition. We worry that in the future some other women in the future will tease her that her body looks different than theirs.”	“The most worrisome aspect of school is the friends at school who tease him because of the urine smell. My child comes home crying complaining to us that other kids were teasing him because of the smell.”

**Family**	**Collaboration benefits**
“Our family said what was the point of raising such a child. After being married for 10 years, we finally got a child and we will surely take care of him. On seeing his condition, I don’t have anything to say but we won’t give up on him even though my family members have not been supportive.”	“It has been a wonderful experience at the camp. We made a whole group of parents who have been coming here for many years now. We all get together in January and even in between sometimes. The kids play together, and we also spend time chatting.”
“When she was born, all the family members said that your daughter should just be killed because of her condition. They told us that we should poison her because of her condition.”	“I have been coming for 12 years now and there hasn’t been a time when I have missed being here for the camp since then.”
“Yes, our family was very supportive. They said to go get this treatment done and not to worry about anything at home when we were gone as they would handle everything at home for us.”	“Yes, we do feel like family here. We feel thankful that the doctors have been helpful in getting our child better. Other family members are like our close relatives now as we have been coming here for 4 years.”
“My family has been very supportive as they are very open-minded people. We live in a joint family with total of 9 members. We sometimes don’t even have to take care of the child as everyone at home takes care of him.”	“We felt more relieved after seeing other girls have the same condition. We felt more relieved that my child isn’t the only that is going to such a rare condition.”
	“We provide encouragement to the parents, especially to the new ones at the camp.”

In 2019, researchers returned to ask about specific themes that emerged the year prior. Interviewees (*n* = 29) echoed what was learned in the previous year regarding family, benefits of the collaboration, trust in the medical team, and their child’s future. Support from family members was further explored and caregivers identified that the primary type of support received was emotional (*n* = 13, 44.8%) Over half (*n* = 19, 65.5%) reported experiencing stigma and a third (*n* = 9, 31%) reported anticipating stigma from their communities. Many families expressed hope for their child’s future (*n* = 12, 41.4%), but also worried about their child’s ability to get married (*n* = 19, 65.5%), have children (*n* = 16, 55.2%), achieve independence (*n* = 7, 24.1%), and obtain complete continence (*n* = 14, 48.3%).

In 2020, researchers surveyed 72 caregivers of children who underwent surgical repair for BEEC. Descriptive information of the cohort is presented in Table 3. Forty-five (62.5%) reported moderate to severe depressive symptoms. Fifteen (20.8%) were referred immediately for counseling due to symptoms of depression and self-reported history of suicidality. Report of emotional wellbeing decreased as number of subsequent repairs increased (*p* = 0.002) for children who underwent primary repair for BEEC (54, 75%). Mean time since first repair for BEEC was 4.6 years. Of those with 5 years or less time since surgery (46, 63.8%), caregivers reported higher anxiety as number of subsequent repairs increased (*p* = 0.043).

**TABLE 3:** Cohort characteristics and mental health indicators.

Characteristics			Total cohort (*n* = 72)
Age at surgery, m			
Median (25th to 75th percentiles)			20 11–50
Sex, No. (%)			
Male			58 80.56%
Female			14 19.44%
Time since first repair, y			
Avg. (St. Dev.)			4.6 2.78
Diagnosis, No. (%)			
Primary bladder exstrophy			45 26.63%
Redo bladder exstrophy			13 7.69%
Primary penopubic epispadias			12 7.10%
Redo penopubic epispadias			2 1.18%
Beck inventory (ranges from 0 to 63)			
Depression inventory summary score			
	0–10	Normal	24 33.33%
	11–16	Mild mood disturbance	16 22.22%
	17–20	Borderline clinical depression	10 13.89%
	21–30	Moderate depression	12 16.67%
	31–40	Severe depression	9 12.50%
	40+	Extreme depression	1 1.39%
Anxiety inventory summary score			
	0–7	Minimal anxiety	40 55.56%
	8–15	Mild anxiety	15 20.83%
	16–25	Moderate anxiety	15 20.83%
	26–63	Severe anxiety	2 2.78%
RAND 36-Item Mental Health Indicators (ranges from 0 to 100)			
Emotional wellbeing average, Avg. (St. Dev.)			64.67 23.43

## Discussion

This study is the first of its kind to assess the impact of BEEC caregiver distress among LMICs, and more specifically, Indian families. The findings are consistent with previous work on caregiver distress in parents of BEEC children in HICs. Mednick et al. found in a cohort of American caregivers of children with BEEC that their level and frequency of stress was comparable with that of parents of children with type 1 diabetes (3). Specifically, these parents were concerned with the long-term impacts of BEEC on their children, including helping with hygiene and uncertainty about the future. These themes are congruent with the themes identified from Indian parents and caregivers who had anxiety about the post-operative experience. The Indian parents were most concerned about their child’s ability to get married, have children, achieve independence, and obtain complete continence.

The India cohort does differ substantially from the studied HIC families in terms of average socioeconomic status and cultural factors that include multigenerational housing and general access to mental health services. These factors contribute to the added familial and community-based sources of anticipated and experienced stigma that the India cohort identified. Specifically, the parents at Civil Hospital spoke about stigma that arose from their villages and classmates at school with support coming from their families and school administrators and employers. Despite these differences, both sets of families had similar primary concerns surrounding overall emotional and physical wellbeing of their children, and this goes to show that parents around the world have the same anxieties about their children when they are born with congenital urological anomalies.

The high levels of caregiver distress that was elucidated from the 2020 mental health instruments is also consistent with the documented higher risk of depression, anxiety, and suicidality that has been reported in studies on the long-term wellbeing of patients with BEEC (16–19). While these studies focused on patients themselves, the effects of BEEC on parental and caregiver wellbeing are equally drastic and important. Additionally, subsequent surgeries were found to increase the risk of emotional distress for caregivers of children with BEEC, and this was exacerbated when patients and caregivers were within 5 years of their initial operation. With recall bias in mind, this effect could also speak to the diminished stress around medical procedures as patients and caregivers have become accustomed to them overtime and as expectations for families are repeatedly set by physicians and the care team.

### Recommendations

With these considerations in mind, future efforts should be made to continue to address the emotional wellbeing and mental health of BEEC patients and caregivers to maximize overall quality of life for this population. It is important to continue to reduce the need for subsequent surgeries by promoting early and primary referrals as opposed to later and after an initial failed closure at another location. Additionally, continuing to focus on and improve post-operative care of these patients in order to prevent complications that would require subsequent procedures should be made a priority. This is all in addition to the need for robust mental health services for this population to prevent the negative ramifications that accompany caregiver and patient distress. It is well documented that BEEC patients experience higher rates of depression, anxiety, and suicidality than their physically healthy peers (1, 16–19), and any efforts that can be made to reduce this would be beneficial to the long-term wellbeing of these patients. And, now that it is known how high the levels of depression and anxiety are among caregivers of BEEC patients, these same services should be applied to them as well.

In order to achieve this, support of all kinds should be increased for the LMIC BEEC patient and caregiver population. The resource scarcity for BEEC compared to other chronic medical conditions and congenital anomalies is a result of the relative rareness of the condition, but these services are still essential for this population. We are grateful that the Government of Gujarat in India is investing in the infrastructure of Civil Hospital in Ahmedabad and providing funding for the free care of BEEC patients and their caregivers. Still, due to the inherent economic challenges of families treated in LMIC settings, the collaboration continues to solicit support to provide necessary support to families seeking care and strengthening the capacity of Civil Hospital to provide them.

While families at the collaboration are incredibly thankful for the surgical care they receive and there have been numerous improvements seen, we acknowledge that there is more work that could be done to improve the long-term holistic wellbeing of BEEC patients in India. The stigma and anxiety experienced by BEEC patients and their caregivers within India requires more specialized and tailored care in addition to the surgical achievement of anatomical and functional integrity. As a result, the inclusion of long-term wellbeing and quality of life should be included as a metric of post-operative success in both HICs and LMICs. This necessitates the inclusion of efforts to continue to reduce the need for subsequent operations in addition to a robust mental health program for patients and caregivers within the delivery of care to better serve this unique patient populations’ needs.

## Data availability statement

The raw data supporting the conclusions of this article will be made available by the authors, without undue reservation.

## Ethics statement

The studies involving human participants were reviewed and approved by the Institutional Ethics Committee of the Civil Hospital, Ahmedabad, and B. J. Medical College. Written informed consent to participate in this study was provided by the participants or their legal guardian/next of kin.

## Author contributions

JF: full access to all of the data in the study and takes responsibility for the integrity of the data and the accuracy of the data analysis. JR, PR, DC, JP-S, PM, AK, DW, AS, RJ, and JF: concept and design and critical revision of the manuscript for important intellectual content. KS, JR, PU, IN, PL, JS, DW, AS, RJ, and JF: acquisition, analysis, and interpretation of data. KS, DW, AS, and JF: drafting of the manuscript. KS and JF: statistical analysis. DC and AS: obtain funding. All authors contributed to the article and approved the submitted version.
